# Piperine inhibits the proliferation of colorectal adenocarcinoma by regulating *ARL3*-mediated endoplasmic reticulum stress

**DOI:** 10.17305/bb.2024.10525

**Published:** 2024-07-06

**Authors:** Chenqu Wu, Yanqing Qian, Jun Jiang, Deming Li, Li Feng

**Affiliations:** 1 Endoscopic Center, Central Hospital of Minhang, Shanghai, China

**Keywords:** Piperine, *ARL3*, endoplasmic reticulum (ER), colorectal adenocarcinoma (COAD), proliferation.

## Abstract

Colorectal adenocarcinoma (COAD) is a significant cause of cancer-related mortality worldwide, necessitating the identification of novel therapeutic targets and treatments. This research aimed to investigate the role of *ARL3* in COAD progression and to explore the effects of Piperine on *ARL3* expression, cell proliferation, epithelial–mesenchymal transition (EMT), and endoplasmic reticulum (ER) stress. Bioinformatics analysis of The Cancer Genome Atlas (TCGA)–COAD, GSE39582, and GSE44861 datasets assessed *ARL3* expression levels. Immunohistochemical data from the Human Protein Atlas (HPA) database confirmed *ARL3* overexpression in COAD. The association of *ARL3* with COAD clinical parameters and prognosis was also examined. COAD cells were treated with Piperine, and in vitro assays evaluated cell proliferation, apoptosis, EMT marker expression, and ER stress (ERS) responses. *ARL3* overexpression in COAD correlated with poor prognosis and varied across pathological stages. Piperine treatment inhibited COAD cell proliferation in a concentration- and time-dependent manner, as indicated by reduced Ki-67 levels and decreased colony-forming ability. Piperine induced S-phase cell cycle arrest and facilitated apoptosis in COAD cells, evidenced by changes in Bax, Bcl-2, cleaved caspase-3, and cleaved Poly (ADP-ribose) polymerase (PARP) levels. Moreover, Piperine downregulated *ARL3* expression in COAD cells, thereby suppressing transforming growth factor beta (TGF-β)-induced EMT. Additionally, Piperine attenuated the *ARL3*-mediated ER stress response, significantly reducing binding immunoglobulin protein (BiP), inositol-requiring enzyme 1 alpha (p-IRE1α), activating transcription factor 6 (ATF6), and C/EBP homologous protein (CHOP) levels. Piperine exerts anti-cancer effects in COAD by modulating *ARL3* expression, disrupting cell cycle progression, inhibiting the EMT pathway, and regulating ERS. These findings suggest that Piperine holds promise as a therapeutic agent for COAD through its targeting of *ARL3*.

## Introduction

Colorectal adenocarcinoma (COAD) is the most prevalent type of colon cancer (CC) tumor, characterized by high incidence and potential fatality [1]. In the early stages, there are no obvious symptoms, but in the late stages, it may cause abdominal pain, blood in the stool, and weight loss [2]. Its etiology involves a multifactorial interplay of genetic, environmental, and lifestyle factors. Contributing factors include age, family history of COAD, sedentary lifestyle, dietary habits, smoking, obesity, and excessive binge drinking [3]. COAD accounts for a significant global proportion of cancer-related incidence and mortality. Even with improvements in early diagnosis and therapeutic approaches, the survival rates for COAD remain suboptimal, particularly in the advanced stages of the disease [4]. Current therapeutic approaches for COAD include immunotherapy, targeted treatment, radiation therapy, chemotherapy, and surgery [5]. However, the heterogeneity and complexity of COAD result in widely varying treatment outcomes and high relapse rates, highlighting the need for continued research into its pathogenesis, early detection, and novel treatments.

Piperine, a natural alkaloid derived from black pepper, exhibits diverse biological functions, such as antioxidant, anti-inflammatory, and anticancer effects [6]. Piperine is characterized by its safety and stability [7]. Its ability to modulate drug-metabolizing enzymes and enhance intestinal absorption underscores its potential for therapeutic applications. Piperine has been documented to enhance the bioavailability of various therapeutic drugs as well as phytochemicals through this property. In addition, the piperine analogue PGP-41 can inhibit cell resistance to therapeutic drugs [8]. Piperine’s bioavailability-enhancing property is also partly attributed to increased absorption resulting from its effect on the ultrastructure of the intestinal brush border [9]. Notably, studies have highlighted piperine’s role in COAD research. Current studies have shown that piperine can inhibit colorectal cancer by regulating STAT3/Snail-mediated epithelial–mesenchymal transition (EMT), Nrf-2/Keap-1, and Wnt/β-catenin pathways [10–12], indicating that piperine may affect colorectal cancer through multiple signaling pathways. Yüksel et al. [13] demonstrated its ability to amplify the anti-tumorigenic effects of cannabinoids and curcumin against COAD cells. Additionally, research by Yaffe et al. [14] revealed that piperine restrains the proliferation of human COAD cells by triggering G1 arrest and apoptosis through endoplasmic reticulum (ER) stress. Moreover, Srivastava et al. [15] found that Piperine enhances the anti-proliferative effects of celecoxib on COAD cells by modulating the Wnt/β-catenin signaling pathway. Such findings underscore the significance of Piperine in COAD research, indicating that it might be an effective therapeutic target in this area.

*ARL3*, a small GTPase of the ADP-ribosylation factor family, plays critical roles in intracellular trafficking, cytoskeletal organization, and ciliary function [16]. Dysregulation of *ARL3* is implicated in various diseases, including cancer and ciliopathies. Additionally, *ARL3* has been identified as a potential therapeutic target due to its involvement in cell proliferation, migration, and differentiation. Studies by Zhang et al. [17] revealed that *ARL3* mutations are closely associated with ciliopathies, impacting cilia morphology and function. Furthermore, research conducted by Rao et al. [18] demonstrated that Circ-*ARL3* serves as a crucial regulator in hepatitis B virus-related hepatocellular carcinoma by sponging miR-1305, thus facilitating cancer progression. Moreover, findings from Wang et al. [19] showed that *ARL3* is downregulated and functions as a prognostic biomarker in glioma, suggesting its potential involvement in antiangiogenesis and the invasion of immune cells within the tumor tissue environment. These findings underscore the importance of further investigating *ARL3* to gain valuable insights into its biological functions and implications in disease mechanisms. However, the specific role of *ARL3* in COAD has not been extensively studied. Preliminary data suggest that *ARL3* might influence cancer cell behavior, possibly through modulating signal transduction pathways or interacting with other proteins involved in tumor progression. Understanding the function of *ARL3* in the context of COAD could reveal novel mechanisms of tumorigenesis and identify potential therapeutic targets. This study aims to fill this gap by investigating the expression and functional implications of *ARL3* in COAD, thereby providing new insights into its potential role in colorectal cancer progression.

This study characterized the mechanistic function of *ARL3* in COAD progression using bioinformatics approaches and in vitro analyses. In parallel, it evaluated the efficacy of Piperine, an alkaloid extracted from black pepper, as a modulator of *ARL3* expression and an antagonist of proliferation, invasion, and migration behaviors in a COAD cell model. The study scrutinizes the effects of Piperine on cell cycle dynamics, apoptotic processes, ER stress (ERS) responses, and EMT. In conclusion, it highlights the prognostic significance of *ARL3* and the therapeutic potential of Piperine in COAD. By targeting *ARL3* and modulating ERS pathways, Piperine offers a promising approach to inhibit tumor progression. While further research is needed to fully elucidate the role and clinical relevance of *ARL3* and Piperine, our findings provide a strong foundation for future investigations into innovative strategies for COAD therapy.

## Materials and methods

### Screening of COAD-related differentially expressed genes (DEGs) in public databases

Microarray datasets were retrieved from the Gene Expression Omnibus (GEO, https://www.ncbi.nlm.nih.gov/gds/) and The Cancer Genome Atlas (TCGA, https://tcga-data.nci.nih.gov/tcga) [20, 21]. The TCGA-COAD database comprised 455 COAD samples and 41 normal samples. The GSE39582 dataset comprised 566 CC samples and 19 normal samples, and the GSE44861 dataset comprised 56 CC samples and 55 normal samples. Probe IDs from these datasets were translated into gene symbols, ensuring consistency and comparability across different datasets. Preprocessing was performed using the R programming language with the Limma package for differential analysis. The standard for differential analysis was set as a fold change (FC) threshold > 1.3; 1.3 for upregulated DEGs, FC < 0.77 for downregulated DEGs, and *P* value < 0.05. These thresholds were chosen to balance the discovery of meaningful gene expression changes while controlling for false positives.

### Assessment of ARL3 expression and its prognostic significance in COAD

*ARL3* expression in COAD samples and normal control samples from the TCGA-COAD dataset, GSE39582 dataset, and GSE44861 dataset was assessed using Wilcox tests. This statistical analysis was employed to contrast the expression levels of *ARL3* between COAD samples and normal controls in each dataset. Additionally, Kaplan–Meier (KM) survival analysis was performed to assess the impact of differential *ARL3* expression on overall survival (OS) probability in COAD patients. For this analysis, patients were stratified into high and low-expression groups based on the median *ARL3* expression level within each dataset. The log-rank *P* value was calculated to assess the significance of the observed differences in OS probability between groups stratified based on *ARL3* expression levels. The survival curves were visualized to provide a clear depiction of the survival probabilities over time for each group.

### Exploring protein levels of ARL3 in the Human Protein Atlas (HPA) database

A large amount of transcriptome and proteome data are available for free online in the Human Protein Atlas (HPA v18.1) database (https://www.Proteinatlas.org/). In this study, immunohistochemical analysis of COAD tumor tissue and adjacent normal colon tissue in this database was performed. *ARL3* expression staining was performed using a validated anti-*ARL3* antibody (Cat. No. HPA036292). Staining intensity was quantitatively assessed and classified as “high,” “medium,” or “low” to analyze the comparison of *ARL3* expression between tumor tissue and normal tissue.

### Comparison of clinical information on *ARL3* expression in COAD

Based on the data of 455 COAD samples provided by the TCGA database, we used the Assistant for Clinical Bioformation platform (https://www.aclbi.com/static/index.html#/tcga) to deeply study the role of *ARL3* in COAD expression characteristics. Our analysis covered a variety of clinical parameters, including patient status, race, gender, pT stage, pN stage, pM stage, and pTNM stage, to understand the differential expression of *ARL3* on different clinical parameters of COAD. When *P* < 0.05, the results obtained are statistically significant.

### Cell lines and culture

COAD cells, including HCT116 and HT29, were obtained from the Cell Bank of the Chinese Academy of Sciences (Shanghai, China) and maintained in Dulbecco’s Modified Eagle Medium (DMEM, Gibco, USA) supplemented with 10% fetal bovine serum (FBS, Gibco, USA) along with 1% penicillin–streptomycin. The temperature of the cell cultures was maintained at 37 ^∘^C in a humidified environment with 5% CO_2_.

### Cell treatment and transfection

Piperine is a natural alkaloid found in black pepper that drives cancer cell death. Piperine (Sigma-Aldrich, German) was dissolved in dimethyl sulfoxide (DMSO) to prepare stock solutions, which were directly added to the cell culture medium. Cells with only DMSO were added as the control group (Control). Six-well plates were used to seed the COAD cells, and they were treated with 25, 50, 100, and 150 µM piperine for 12, 24, 36, 48, 60, and 72 h for induction [10, 12]. In a parallel experiment, to induce EMT, COAD cells underwent treatment using 10 ng/mL TGF-β (Sigma, USA) for 48 h. For gene overexpression studies, a seeding density of 2 × 10^5^ cells/well was used to sow cells in 24-well plates for transient transfection experiments. Transfection of cells was done using plasmid encoding *ARL3* using Lipofectamine 3000 reagent (Invitrogen, Shanghai, China) based on the guidelines provided by the supplier to promote *ARL3* overexpression and evaluate its biological effects in transfected COAD cells.

### Quantitative real-time polymerase chain reaction (qRT-PCR)

The total RNA of COAD cells was extracted using the TRIzol reagent (Tiangen, Beijing, China) as directed by the manufacturer. For complementary DNA (cDNA) synthesis, we utilized a PrimeScript RT kit from Dalian, China. The reverse transcription process was carried out following the manufacturer’s instructions to convert the RNA into cDNA. qRT-PCR was performed using SYBR Green PCR Master Mix (Takara, China) on the StepOnePlus Real-Time PCR System (Applied Biosystems, Shanghai, China). Each sample was run in triplicate to ensure reproducibility. The 2^−ΔΔCT^ technique was employed to analyze the results, and β-actin abundance served as a standard. The following primer sequences were used in the amplification process: *ARL3* forward: 5′-GGACAGAGGAAAATCAGACCATACT-3′, *ARL3* reverse: 5′-GTCGCGGATGGTATGCAGGT-3′. Similarly, the forward and reverse primers for β-actin used as the reference gene were: β-actin forward: 5′-GTTGCTATCCAGGCTGTG-3′, β-actin reverse: 5′-TGATCTTGATCTTCATTGTG-3′.

### Western blot (WB) assay

Protease and phosphatase inhibitors (CoWin Biosciences, Nanjing, China) were added to the RIPA lysis buffer (Solarbio, Beijing, China) to create protein lysates from COAD cells. Cells were harvested and lysed on ice to ensure complete protein extraction while minimizing degradation. The BCA Protein Assay Kit (Beyotime, China) was used to measure the protein concentration. Proteins in equal quantities were separated using 10% SDS-PAGE and then transferred onto PVDF membranes from Beyotime in Beijing, China. After blocking the membrane with 5% skim milk in Tris-buffered saline with Tween-20 (TBST) for 1 h at room temperature to prevent nonspecific binding, the primary antibody was diluted 1:1000 in TBST containing 5% BSA and incubated overnight at 4 ^∘^C. Primary antibodies included anti-Ki-67, anti-cyclin D1, anti-CDK6, anti-p27, anti-Bax, anti-caspase 3, anti-cleaved caspase 3, anti-PARP, anti-cleaved PARP, anti-Bcl-2, anti-E-cadherin, anti-Snail, anti-N-cadherin, anti-BIP, anti-IRE1α/p-IRE1α, anti-ATF6 (Abcam, China), anti-*ARL3*, and anti-CHOP (Wuhan Sanying Biotechnology Co., Ltd., Wuhan, China). Following primary antibody incubation, the appropriate horseradish peroxidase-conjugated secondary antibodies were used to probe the membranes. Protein bands were visualized using an enhanced chemiluminescence (ECL) kit and captured with a ChemiDoc imaging system (Bio-Rad, Shanghai, China). The intensity of the bands was quantified using ImageJ software to ensure accurate and reproducible results.

### Cell counting kit-8 (CCK-8) assay

The CCK-8 assay (KeyGEN, Nanjing, China) was utilized to evaluate the viability of the cells. In 96-well plates, COAD cells were seeded at a density of 5 × 10^3^ cells per well. At designated time points of 12, 24, 36, 48, 60, and 72 h post-treatment, 10 µL of CCK-8 reagent was added to each well. Following the addition of the CCK-8 reagent, the plates were incubated at 37 ^∘^C for 1–2 h to allow for the development of the colorimetric signal. A microplate reader (Kehua Technologies, Inc., Shanghai, China) was employed to measure the absorbance at 450 nm.

### Colony formation assay

The capacity of the cells to form colonies was assessed using a colony formation test. Briefly, 2000 cells were planted in 60-mm plates and cultured for two weeks at 37 ^∘^C with 5% CO_2_. After fixation with methanol for 30 min, the cells were stained with nitro blue tetrazolium chloride overnight. Images were taken using a Gel imaging system (Hangzhou Shenhua Technology Co., Ltd., Hangzhou, China), and ImageJ Software version 1.53t was used to count the colonies. Colonies containing more than 50 cells were tallied. Each experiment was run three times to ensure the reproducibility and reliability of the results.

### Flow cytometry

For flow cytometry analysis, COAD cells were detached using trypsin-EDTA (Life Technologies Inc., Beijing, China) and rinsed with phosphate-buffered saline (PBS). Cells were then resuspended in a binding buffer and stained with Annexin V and propidium iodide (PI) to differentiate viable, apoptotic, and necrotic cells according to the manufacturer’s instructions. The staining process was carried out at room temperature in the dark. Flow cytometry was performed using a flow cytometer (Jiyuan, Guangzhou, China), and data were assessed using FlowJo software (FlowJo, Hangzhou, China) to quantify the cell apoptosis rate. Cell cycle assays were conducted by plating cells in 6-well plates at a density of 1×10^5^ cells/well and cultured overnight at room temperature. Following this, the cells were exposed to PI and RNase A at 37 ^∘^C for 40 min. Subsequently, the distribution of cells in the G1, S, and G2 phases of the cell cycle was determined by analyzing the cell samples using flow cytometry.

### Cell invasion and migration assays

Transwell was used to assess cell invasion and migration. The upper chamber of the Transwell contained suspended transfected COAD cells in a serum-free medium, while 10% FBS was introduced into the medium within the lower compartment of the Transwell. After an incubation period, DAPI was used to stain cells for 30 min with moving cell membranes after they had been treated with 4% paraformaldehyde for 20 min at room temperature. The quantity of migratory cells in the field of view was recorded using inverted microscopy. Cell invasion studies were carried out as described previously, with matrigel covered in the upper chamber.

### Statistical analysis

Statistical analysis was performed using the R program with the R package “Limma.” Comparisons between two independent groups were done using the student’s *t*-test. For multiple group comparisons, a one-way ANOVA was paired with the Tukey post hoc test. The non-parametric paired data analysis was conducted using the Wilcoxon matched pairs signed rank test. For statistical significance, the threshold was established at *P* < 0.05, and data were shown as mean ± standard deviation (SD).

## Results

### Association of ARL3 expression with prognosis and clinicopathological parameters in COAD

**Figure 1.**
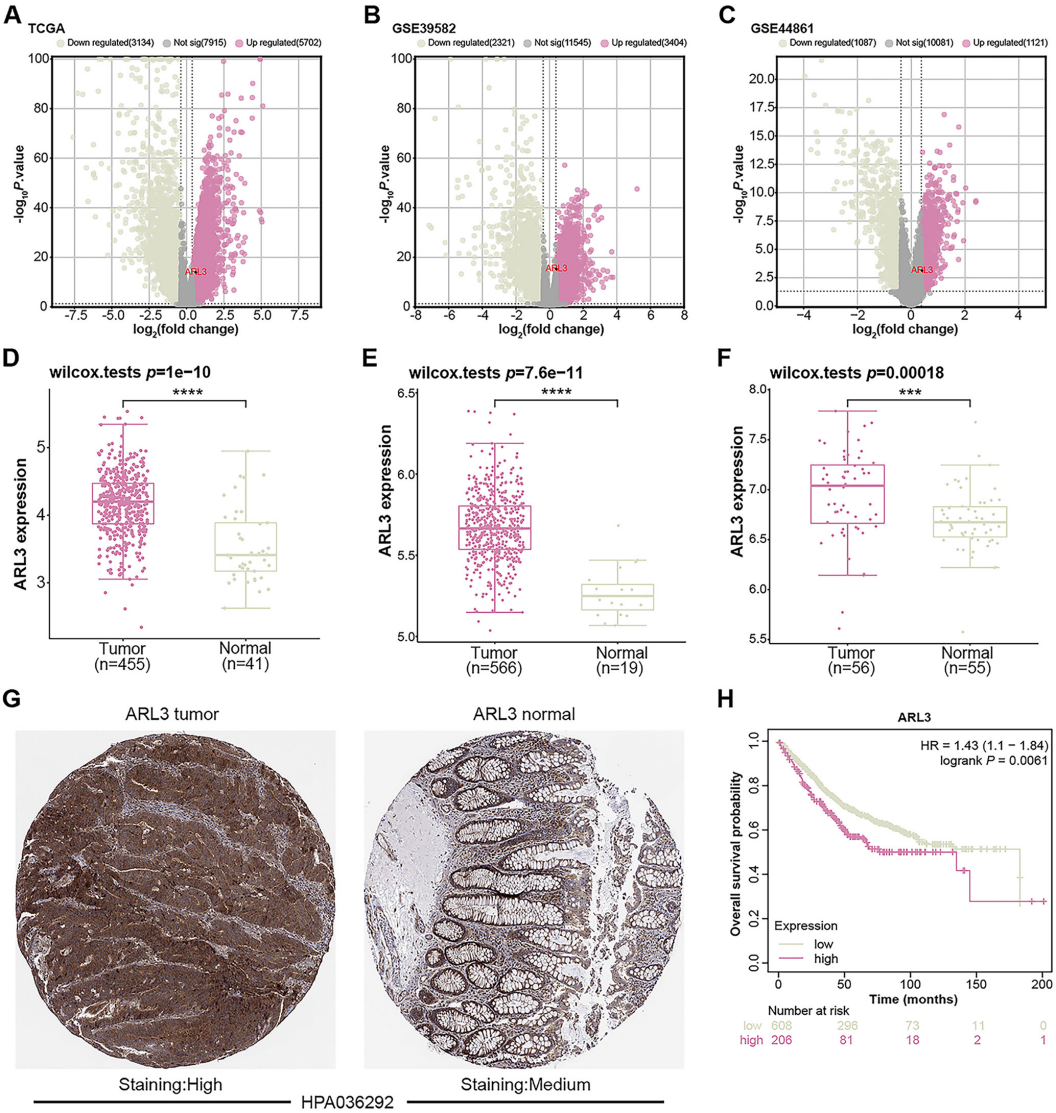
**Identification of DEGs in COAD and the prognostic significance of *ARL3*.** (A–C) DEGs screening of TCGA-COAD dataset, GSE39582 dataset, and GSE44861 dataset. Red represents upregulated DEGs, green represents downregulated DEGs, and gray represents insignificant genes; (D–F) Expression of *ARL3* in tumor samples and normal samples in the TCGA-COAD dataset, GSE39582 dataset, and GSE44861 dataset. Red represents tumor samples, and green represents normal samples; (G) Immunohistochemical staining of *ARL3* in COAD detected by HPA database. The left panel (*ARL3* tumor) shows a representative tissue section from a COAD showing high staining intensity, and the right panel (*ARL3* normal) shows a section from adjacent normal colon tissue with moderate staining intensity. Catalog number HPA036292 refers to the antibody used for staining; (H) OS prognosis of high *ARL3* expression and low *ARL3* expression. Red represents high expression and green represents low expression. ***P < 0.001, ****P < 0.0001. COAD: Colorectal adenocarcinoma; DEGs: Differentially expressed genes; TCGA: The Cancer Genome Atlas; OS: Overall survival prognosis; HPA: Human Protein Atlas.

**Figure S1.**
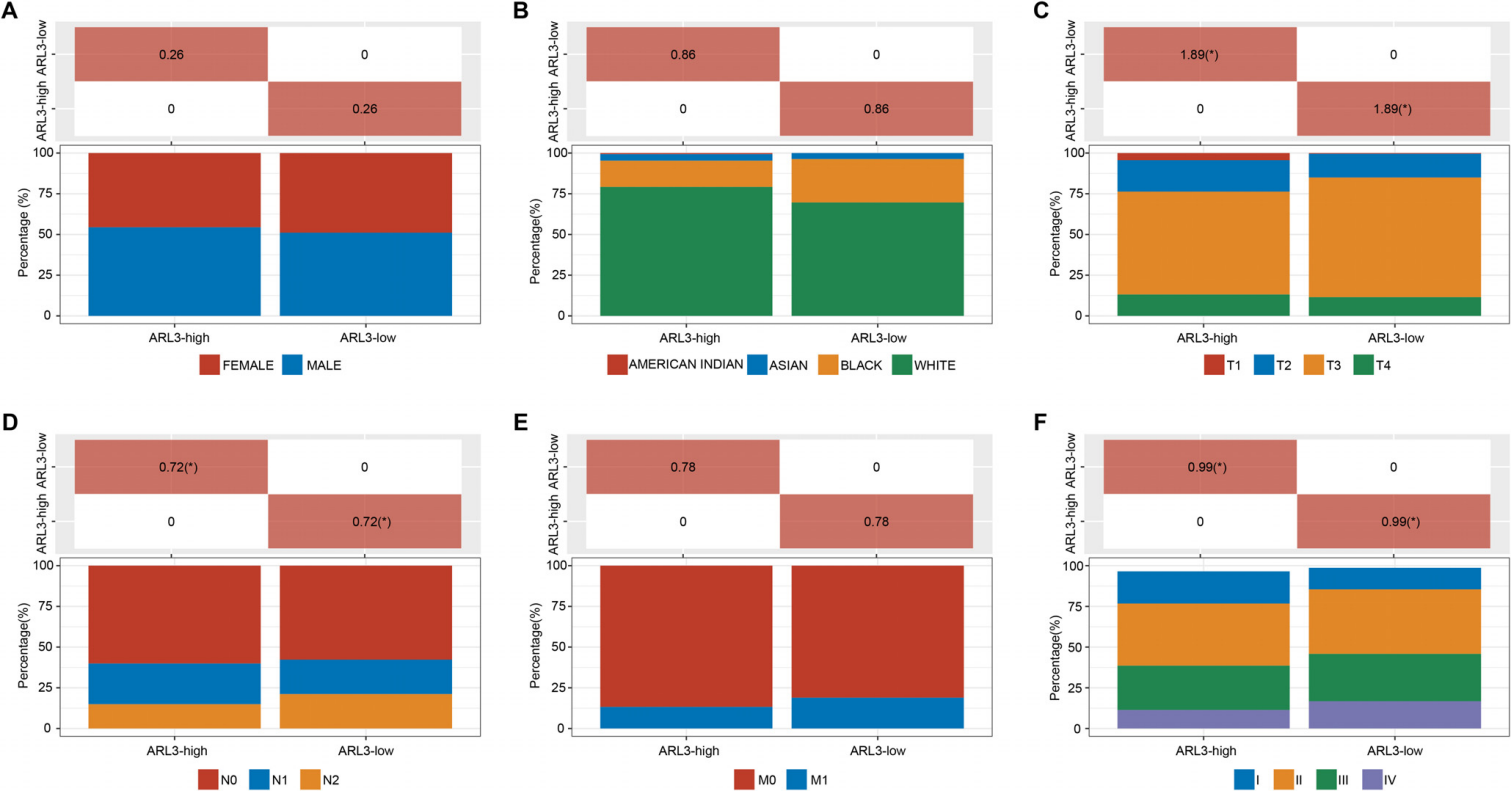
**Comparison of characteristics of the COAD patients with high and low *ARL3* expression.** (A) The percentage of male and female patients in *ARL3* high and low expression samples; (B) The percentage of different races in high/low gene expression samples; (C) The percentage of different pT stages in high/low gene expression samples; (D) The percentage of different pN stages in high/low gene expression samples; (E) The percentage of different pM stages in high/low gene expression samples; (F) The percentage of different pTNM stages in high/low gene expression samples.

Using the R package, 5702 upregulated DEGs and 3134 downregulated DEGs were identified from tumor and normal samples obtained from the TCGA database. Additionally, 3404 upregulated DEGs and 2321 downregulated DEGs were identified from the GSE39582 dataset, while 1121 upregulated DEGs and 1087 downregulated DEGs were identified from the GSE44861 dataset (Figure 1A–1C). Among them, *ARL3* was within the range of up-regulated DEGs in these three datasets. Subsequent Wilcoxon tests revealed significant overexpression of *ARL3* in tumor samples from the TCGA-COAD dataset, GSE39582 dataset, and GSE44861 dataset (Figure 1D–1F). Immunohistochemical analysis of the HPA database showed markedly elevated expression of *ARL3* in COAD tumor tissue compared with adjacent normal colon tissue (Figure 1G). KM survival assay revealed that elevated expression of *ARL3* resulted in a worse patient prognosis (Figure 1H). Furthermore, clinical expression analysis demonstrated a substantial correlation between the diseased stage and the differential expression of *ARL3* (pT, pN, and pTNM) in COAD patients (Figure S1 and Table S1). This suggests that *ARL3* expression may serve as a predictive measure of COAD progression.

### Piperine inhibits COAD cell proliferation by inducing cell cycle arrest

**Figure 2.**
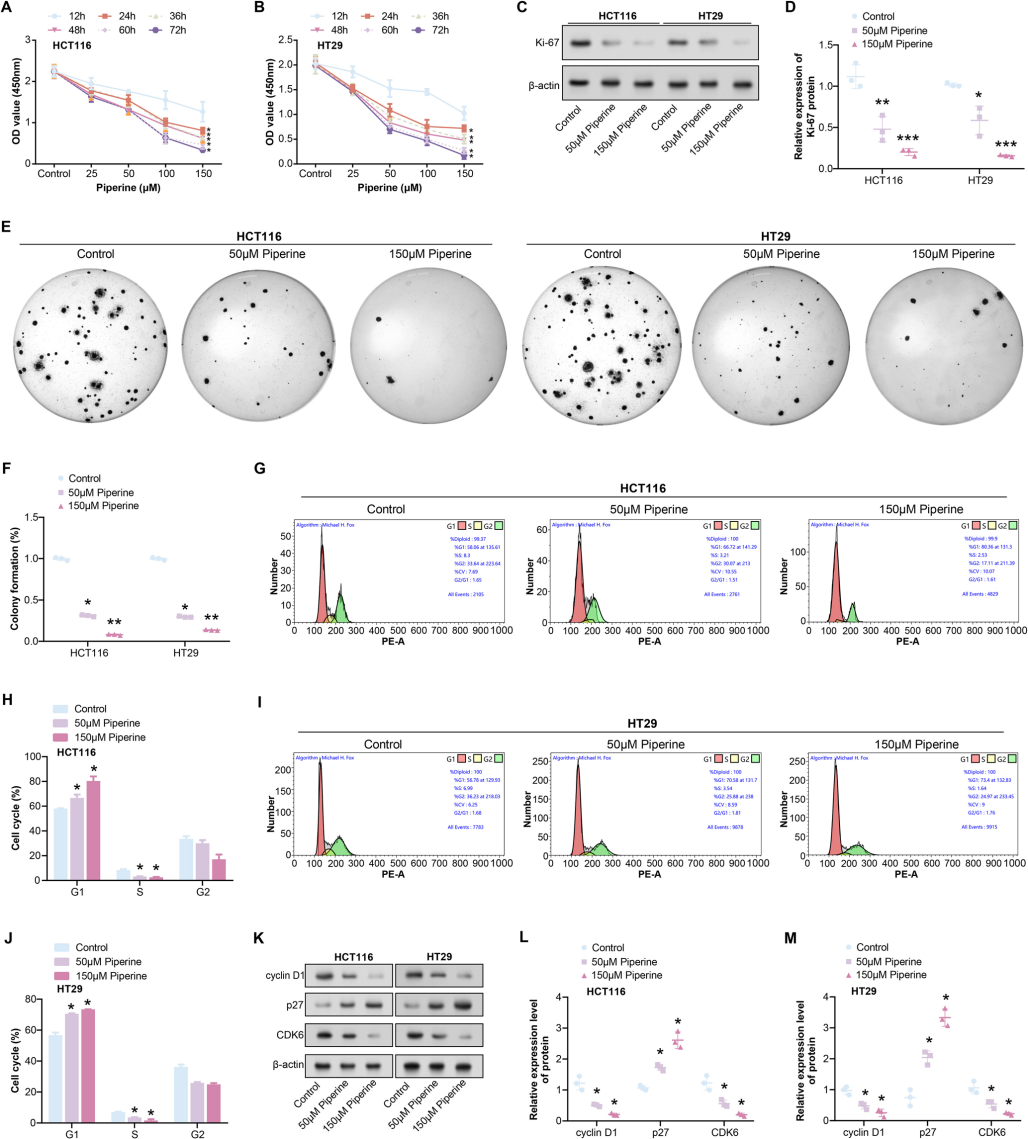
**Piperine inhibits COAD cell proliferation by inducing cell cycle arrest.** (A and B) CCK-8 detected changes in the proliferation ability of COAD cells (HCT116 and HT29) after induction with different concentrations of Piperine at different times; (C and D) WB detected changes in the expression level of Ki-67 protein in HCT116 and HT29 cells after treatment with 50 and 150 µM Piperine; (E and F) A colony formation assay was used to detect the effects of 50 µM Piperine and 150 µM Piperine on the proliferation of HCT116 and HT29 cells for 48 h, and quantitative analysis was performed; (G–J) Flow cytometry detected cell cycle changes after COAD cells are treated with 50 µM Piperine and 150 µM Piperine; (K–M) WB detection of the effects of 50 µM Piperine and 150 µM Piperine treatment on COAD cell cycle proteins. *P < 0.05, **P < 0.01, ***P < 0.001. COAD: Colorectal adenocarcinoma; CCK-8: Cell counting kit-8; WB: Western blot.

Piperine, a natural alkaloid found in black pepper, has been demonstrated to induce cancer cell death. CCK-8 assays revealed a significant decrease in the proliferation capability of COAD cells (HCT116 and HT29) with increasing concentrations and induction times of piperine (Figure 2A and 2B). WB analysis detected a significant decrease in Ki-67 (cell proliferation marker) protein expression in HCT116 and HT29 cells after treatment with 50 µM Piperine, with a further decrease observed after treatment with 150 µM Piperine (Figure 2C and 2D). Similarly, colony formation assays confirmed the decrease in colony formation of COAD cells after treatment with 50 µM Piperine, with a more pronounced reduction observed after treatment with 150 µM Piperine (Figure 2E and 2F). Flow cytometry analysis showed a cell cycle halt at the S phase in COAD cells after Piperine treatment (Figure 2G–2J). WB analysis of cell cycle protein expression in COAD cells showed that treatment with 50 µM Piperine reduced the levels of cyclin D1 and CDK6 proteins, which were further enhanced by treatment with 150 µM Piperine. Additionally, treatment with 50 µM Piperine increased the expression of p27, which was further enhanced by treatment with 150 µM Piperine (Figure 2K–2M).

### Piperine induces apoptosis in COAD cells

**Figure 3.**
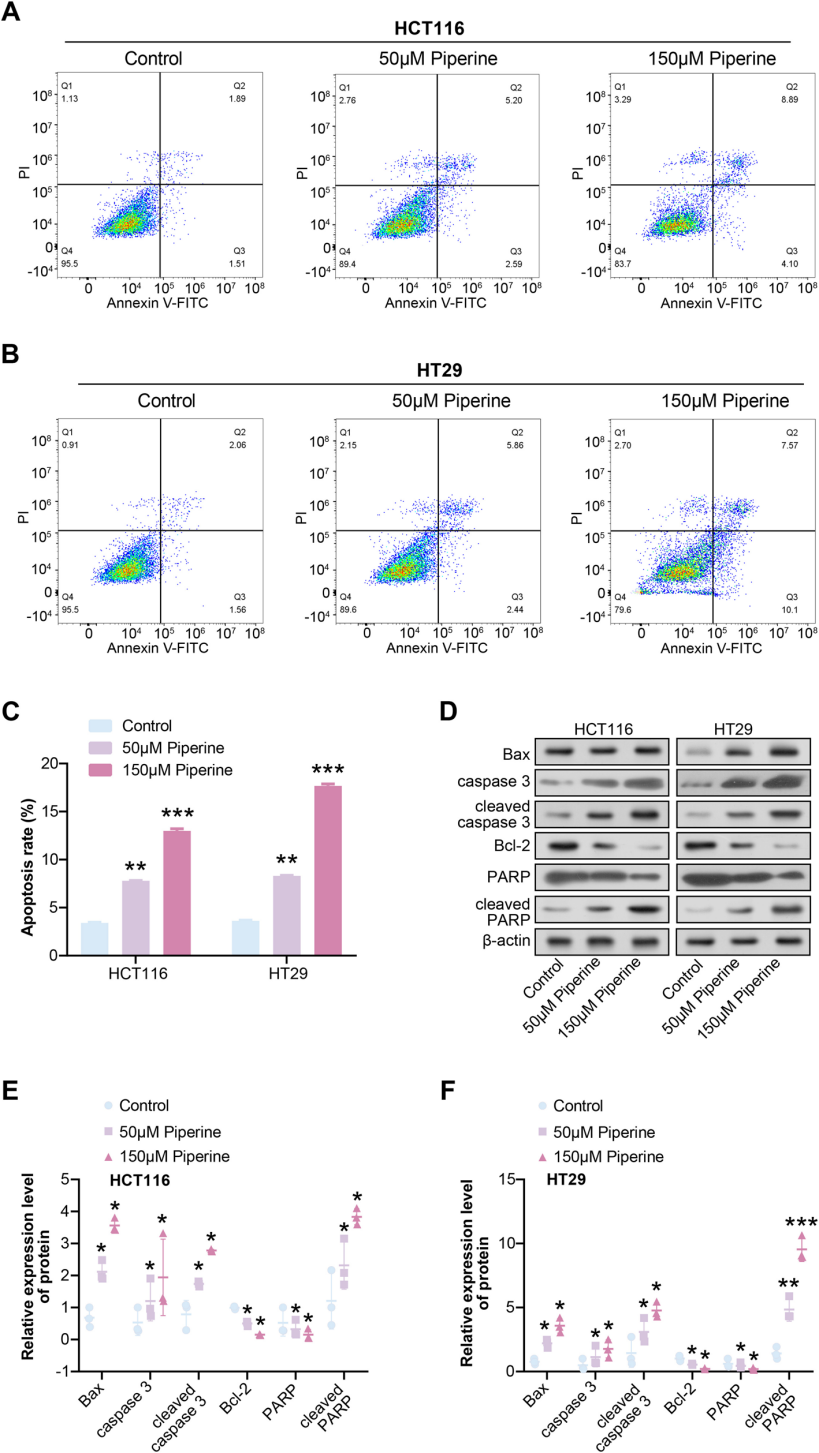
**Piperine induces apoptosis in COAD cells.** (A–C) Flow cytometry was used to detect the effects of 50 µM Piperine and 150 µM Piperine on COAD cell apoptosis after 48 h of treatment, and quantitative analysis was performed; (D–F) WB detection of the effects of 50 µM Piperine and 150 µM Piperine on apoptotic proteins (Bax, caspase 3, cleaved caspase 3, Bcl-2, PARP, cleaved PARP) in HCT116 and HT29 cells. **P < 0.01, ***P < 0.001. COAD: Colorectal adenocarcinoma; WB: Western blot.

Analysis using flow cytometry revealed that the apoptosis rate of COAD cells increased significantly after induction with 50 µM Piperine for 48 h, and the addition of 150 µM Piperine further enhanced the apoptosis rate (Figure 3A–3C). This was also confirmed by WB analysis, in which the levels of pro-apoptosis-related proteins (Bax, caspase-3, cleaved caspase-3, and cleaved PARP) in COAD cells were significantly upregulated after induction with 50 µM Piperine, while the levels of Bcl-2 (an apoptosis inhibitory protein) and PARP were significantly reduced. These changes were further enhanced upon induction with 150 µM Piperine (Figure 3D–3F).

### Piperine inhibits ARL3 expression and TGF-**β**-induced EMT

**Figure 4.**
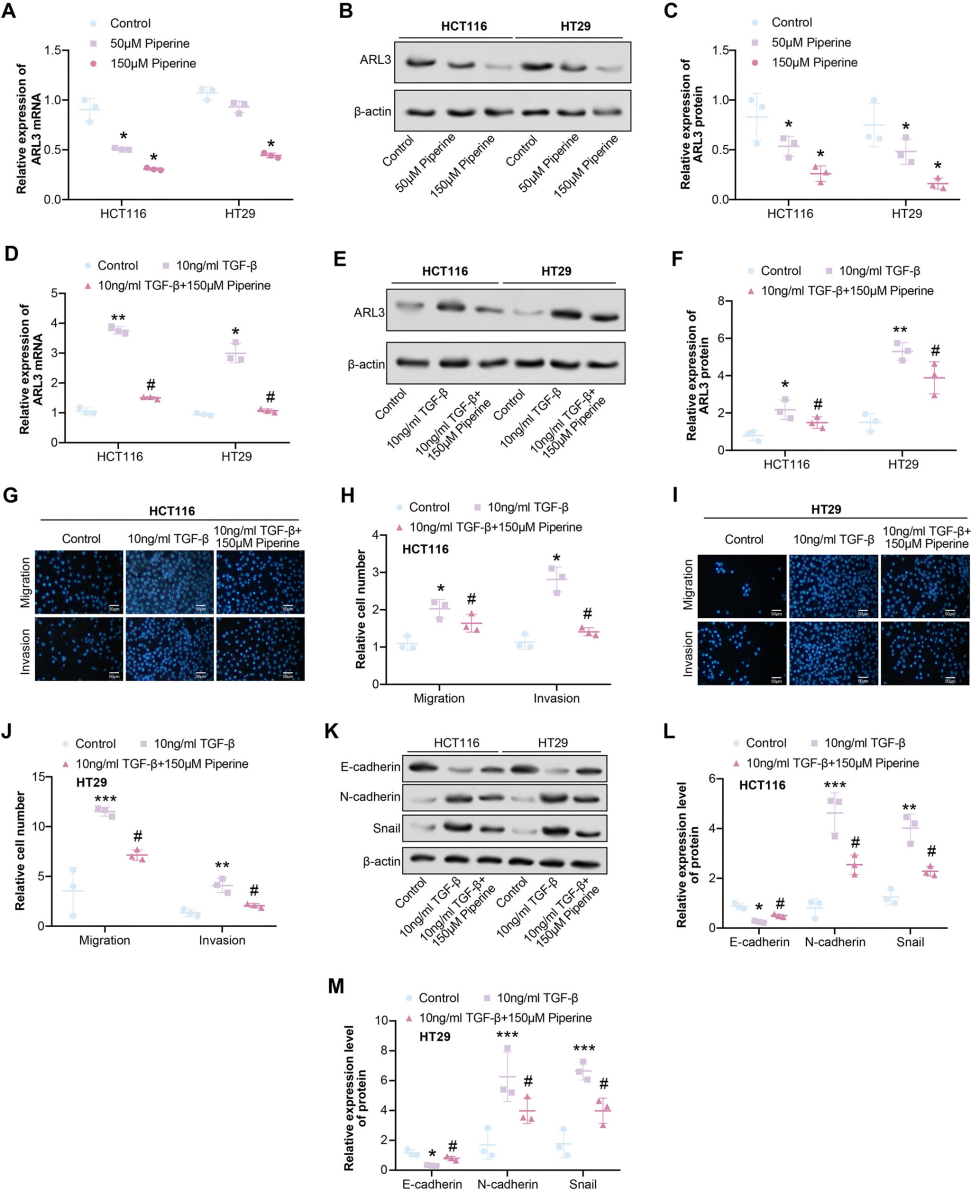
**Piperine inhibits *ARL3* expression and inhibits TGF-β-induced EMT.** (A–C) qRT-PCR and WB detected the expression of *ARL3* after COAD cells were treated with 50 µM Piperine and 150 µM Piperine; (D–F) qRT-PCR and WB detected the expression of *ARL3* in COAD cells after treatment with 10 ng/mL TGF-β and 150-µM Piperine; (G–J) Transwell was used to detect the changes in cell invasion and migration ability after COAD cells were treated with 10 ng/mL TGF-β and 150 µM Piperine for 48 h, and quantitative analysis was performed; (K–M) WB analysis of expression changes of EMT marker proteins in COAD cells treated with 10 ng/mL TGF-β and 150 µM Piperine for 48 h. *P < 0.05 or **P < 0.01 vs Control group, ^#^*P* < 0.05 vs 10 ng/mL TGF-β group. COAD: Colorectal adenocarcinoma; qRT-PCR: Quantitative real-time polymerase chain reaction; WB: Western blot; EMT: Epithelial–mesenchymal transition.

qRT-PCR and WB analyses revealed that after 48 h of induction with 50 µM Piperine, the expression levels of *ARL3* in COAD cells decreased, with a more pronounced decrease observed under 150 µM Piperine induction (Figure 4A–4C). Furthermore, qRT-PCR and WB analyses showed that stimulation with 10 ng/mL TGF-β significantly increased the expression levels of *ARL3* in COAD cells. However, co-treatment with 150 µM Piperine attenuated this upregulation (Figure 4D–4F). Similarly, transwell assays demonstrated that TGF-β induction at 10 ng/mL significantly enhanced the migration and invasion abilities of COAD cells, while this effect was significantly attenuated after the addition of 150 µM Piperine (Figure 4G–4J). E-cadherin, N-cadherin, and Snail are markers associated with EMT. WB analysis showed that TGF-β treatment significantly reduced E-cadherin levels while increasing N-cadherin and Snail protein levels, indicating EMT activation. The introduction of 150 µM Piperine effectively counteracted these TGF-β-induced changes in protein levels (Figure 4K–4M).

**Figure 5.**
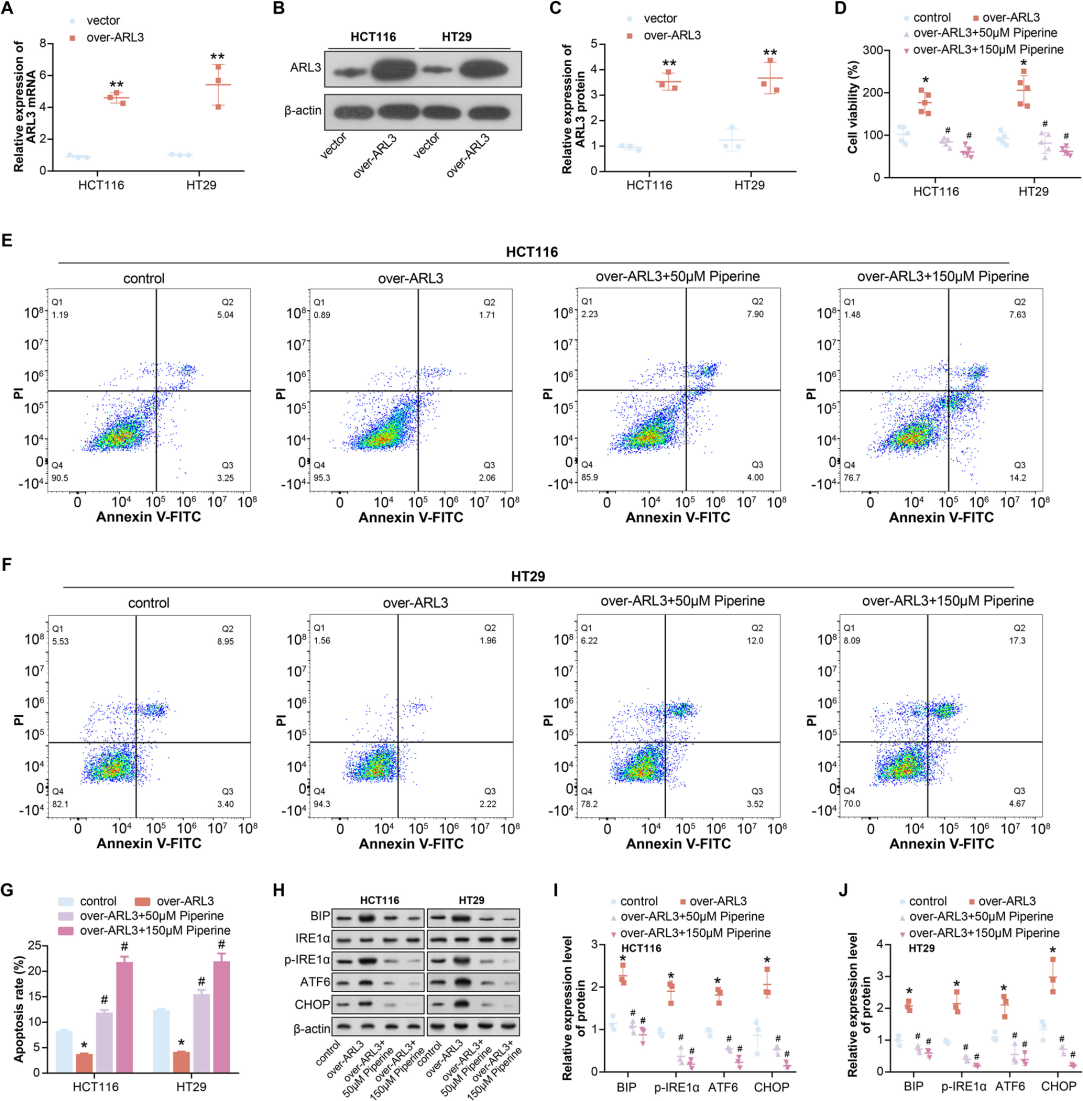
**Piperine promotes overexpression of *ARL3* and antagonizes ER stress.** (A–C) qRT-PCR and WB detected the overexpression efficiency of *ARL3* in COAD cells; (D) CCK-8 detection of COAD cell proliferation in different treatment groups. The groups included: Control, over-*ARL3*, over-*ARL3*+50 µM Piperine, and over-*ARL3*+150 µM Piperine; (E–G) Flow cytometry was used to detect the apoptosis rate of COAD cells in different treatment groups. The groups included: Control, over-*ARL3*, over-*ARL3*+50 µM Piperine, and over-*ARL3*+150 µM Piperine; (H–J) WB detection of changes in ER stress-related proteins (BIP, IRE1α, p-IRE1α, ATF6, CHOP) in COAD cells in different treatment groups. The groups included: Control, over-*ARL3*, over-*ARL3*+50 µM Piperine, and over-*ARL3*+150 µM Piperine. *P < 0.05 or **P < 0.01 vs control group, ^#^*P* < 0.05 vs over-*ARL3* group. COAD: Colorectal adenocarcinoma; qRT-PCR: Quantitative real-time polymerase chain reaction; WB: Western blot; ER: Endoplasmic reticulum; CCK-8: Cell counting kit-8.

### Piperine promotes overexpression of ARL3 and antagonizes ERS

Initially, qRT-PCR and WB analyses demonstrated efficient overexpression of *ARL3* in COAD cells (Figure 5A–5C). Subsequently, CCK-8 and flow cytometry analyses revealed that *ARL3* overexpression considerably increased cell proliferation and viability while decreasing the apoptosis rate in COAD cells. However, the addition of Piperine reversed these changes, with 150 µM Piperine showing a more pronounced reversal (Figure 5D–5G). BIP, p-IRE1α, ATF6, and CHOP are proteins associated with ERS. WB analysis of their protein levels showed that *ARL3* overexpression significantly increased their expression levels; however, the addition of piperine reversed this increase, with 150 µM Piperine showing a more pronounced reversal (Figure 5H–5J). This indicates that piperine affects oxidative stress caused by *ARL3* overexpression.

## Discussion

Current diagnostic modalities of COAD include colonoscopy, computed tomography (CT) scans, and biopsies, each with its limitations in terms of sensitivity and specificity [22]. While surgical resection remains the primary treatment for localized COAD, targeted gene therapies have emerged as promising strategies for advanced cases, aiming to inhibit specific genetic mutations driving tumorigenesis [23]. However, despite advancements in treatment modalities, the five-year survival rate for COAD patients remains suboptimal, emphasizing the urgent need for novel approaches to improve patient outcomes. Biomarkers hold immense potential in revolutionizing the management of COAD by providing valuable insights into disease diagnosis, prognosis, and treatment response [24]. By identifying molecular signatures associated with COAD progression and therapeutic resistance, biomarkers offer the prospect of personalized medicine tailored to individual patients. Bioinformatics analysis of multiple datasets (TCGA-COAD, GSE39582, and GSE44861) indicated that *ARL3* was significantly highly expressed in COAD and linked to poor prognosis. The Wilcoxon test validated the connection of *ARL3* expression with advanced pathological stages, highlighting its potential as a predictive indicator and treatment target in COAD. This finding underscores the importance of further investigating *ARL3* to gain valuable insights into its biological functions and implications in disease mechanisms.

Piperine has a variety of pharmacological properties and is known for its ability to enhance the bioavailability of other drugs and nutrients by inhibiting drug-metabolizing enzymes in the liver and intestines [25]. Piperine is primarily absorbed in the intestines and undergoes hepatic metabolism through cytochrome P450 enzymes, forming various metabolites that are excreted in urine [26]. In addition, its potential role in causing apoptosis, preventing tumor formation, and limiting cancer cell growth has been studied [27, 28]. In our study, after treating cells with different concentrations of piperine at different times, it was found that cell proliferation activity decreased in a concentration-time-dependent manner. This is consistent with the findings of Duessel et al. [29], that piperine had a significant concentration-dependent antiproliferative effect on CC cells in vitro. We next examined Ki-67, a protein associated with cell proliferation, to further explore the regulatory effects of piperine on COAD cells. Ki-67 is a commonly used biomarker for detecting cell proliferation activity and is expressed throughout the S, G1, G2, and M phases of the cell cycle. A study by Zhu et al. [30] showed that a high-fat diet promotes colon adenoma formation by increasing Ki-67 expression, indicating enhanced cell proliferation. Additionally, research conducted by Zhao et al. [31] revealed that Ki-67 protein expression in the normal colon mucosa of individuals with colorectal tumors is positively correlated with IGF-II levels, suggesting the role of IGF-II in COAD. In addition to this, cell cycle and apoptosis also play crucial roles in determining cell growth and survival. The results of Zulpa et al. [32] indicate that pks+ *Escherichia coli* strains induce cell cycle arrest and apoptosis in COAD cells and may exert anticancer effects by regulating apoptotic mediators and blocking the S phase. The study by Zorofchian Moghadamtousi et al. [33] demonstrated that custard apple leaves induce G1 cell cycle arrest and apoptosis through mitochondria-mediated pathways in COAD cells. Shaheer et al. [34] found that Piperine can induce increased radiosensitivity in CC cell lines (HT-29) by interfering with cancer cell apoptosis. Likewise, in our study, Piperine was found to inhibit the growth of COAD cells by causing apoptosis and cell cycle arrest, suggesting that Piperine might be an effective drug for regulating the cell cycle and inducing apoptosis.

The multifunctional cytokine TGF-β is involved in immunological control, cell proliferation, and differentiation, among other biological functions. It plays a key role in cancer progression by promoting EMT. Through the process of EMT, epithelial cells lose polarity and gain migration and invasion properties, thereby promoting metastasis [35]. TGF-β promotes the EMT process by inducing transcription factors that suppress epithelial markers and enhance the expression of mesenchymal phenotypes, suggesting that TGF-β is a central mediator of the metastatic potential of cancer cells [36]. Research by Zipfel et al. [37] demonstrates that TGF-β1 promotes the upregulation of TGF-α in human colon carcinoma cells, potentially contributing to COAD progression. Additionally, Zhu et al. [38] found that silencing neuropilin-1 (NRP-1) partially reverses TGF-β1-induced EMT in COAD cells, leading to reduced proliferation and migration, highlighting NRP-1 as a promising therapeutic target. Moreover, Zhu et al. [39] discovered that N-glycosylation of CD82 at Asn157 inhibits EMT by suppressing the Wnt/β-catenin pathway, thereby decreasing metastasis in COAD. In our study, Piperine showed an inhibitory effect on *ARL3* expression and counteracted TGF-β-induced EMT in COAD cells. This was specifically manifested by reduced *ARL3* expression, reduced cell migration and invasion, and changes in EMT marker protein levels after treatment with Piperine. This is similar to the results of Piperine inhibiting EMT in AML-12 liver cells [40]. These findings highlight a possible new way for Piperine to inhibit the malignant progression of COAD by targeting the EMT pathway.

ERS refers to a cellular condition that occurs when the ER is overwhelmed by unfolded or misfolded proteins, triggering the unfolded protein response (UPR) [41]. The goal of this stress reaction is to restore normal ER function by halting protein production, enhancing protein folding capacity, and promoting the breakdown of misfolded proteins. However, prolonged or severe ERS can lead to cell dysfunction and apoptosis, contributing to the pathogenesis of various diseases, including cancer, neurodegenerative disorders, and metabolic syndromes. ERS emerges as a pivotal mechanism in various studies on COAD treatment. Zhu et al. [42] revealed that a purified resin glycoside fraction from Pharbitidis Semen (RFP) induces ERS-mediated proptosis in human COAD cells, offering a potential therapeutic avenue. Meanwhile, Zhou et al. [43] demonstrated that osthole triggers ERS and autophagy, leading to apoptosis in HT-29 colorectal cancer cells, indicating a significant link between ERS and COAD progression. Zhong et al. [44] showed that ERS acts protectively against apoptosis induced by the HK2 inhibitor 3-Bromopyruvate acid (3-BP) in COAD cells, proposing it as a promising strategy for combination therapy. Zhu et al. [45] found that curcumin inhibits irinotecan-induced COAD progression by enhancing immunogenic cell death and inducing ERS, further underscoring the therapeutic potential of targeting ERS in COAD treatment. In addition, Guo et al. [46] confirmed that Piperine alleviated ERS-related neurotoxicity due to the expansion of TBP protein polyQ by inducing MANF expression. Based on this, we analyzed the regulatory effect of Piperine on ERS in COAD and found that Piperine can antagonize the promoting effect of ERS after overexpression of *ARL3*, which may help reduce ERS in COAD cells.

Although this study found that Piperine can inhibit ERS mediated by *ARL3* overexpression, showing potential for COAD treatment, we must also acknowledge several limitations. Firstly, we utilized data from the TCGA database for our study. Despite its extensive dataset, TCGA has intrinsic limitations, such as potential biases in patient selection and data quality, which may affect the generalizability of our findings. These limitations must be considered when interpreting the results and their application to broader populations. Furthermore, our experiments were conducted in vitro, which allows for controlled conditions and detailed mechanistic studies but may not fully replicate the complex interactions and environment found in vivo. Additionally, although we selected Piperine concentrations based on previous studies and preliminary experiments, the absorption, metabolism, and bioavailability of Piperine can vary significantly in a biological system. These pharmacokinetic factors must be thoroughly evaluated in animal models and clinical trials to validate our findings. Future research should aim to address these limitations and further elucidate the role of Piperine in regulating *ARL3* in COAD. Furthermore, the current data may not be sufficient to demonstrate the therapeutic effect of Piperine on ERS or the recovery of cellular function after ERS treatment. To further validate the therapeutic effect of Piperine on ERS in COAD, we will measure key markers of ERS and evaluate cellular function after Piperine treatment to ensure the robustness of our research results. To demonstrate the correlation between *ARL3* and the malignancy of COAD, we will further investigate other cancer cells with high expression of *ARL3*. To further investigate the molecular mechanism underlying the connection between Piperine and *ARL3*, we are considering conducting proteomic and transcriptomic analyses on COAD cells with or without the addition of Piperine. This aims to determine whether Piperine affects COAD by modulating *ARL3*. Additionally, prospective studies involving larger and more diverse patient cohorts are needed to validate the clinical relevance of *ARL3* as a biomarker. In vivo studies using animal models can provide valuable insights into the biological functions of *ARL3* and its potential as a therapeutic target. Investigating the molecular mechanisms and signaling pathways through which Piperine regulates *ARL3* is crucial for understanding its role in COAD progression and identifying potential targets for intervention.

## Conclusion

The differential expression of *ARL3* in COAD sheds light on its prognostic significance, with high expression correlating with poorer outcomes. Piperine, through its inhibitory effects on COAD cell proliferation and induction of apoptosis, presents a promising therapeutic avenue. Piperine not only downregulates *ARL3* expression but also mitigates TGF-β-induced EMT, offering a dual mechanism for inhibiting tumor progression. Notably, the antagonistic effect of Piperine on ERS, mediated by *ARL3* overexpression, highlights its role in modulating cellular responses to stress. These findings underscore the potential of targeting *ARL3* and ERS pathways as an effective strategy in COAD therapy. The identification of Piperine as a novel therapeutic agent provides valuable insights into the development of combinatorial approaches for COAD treatment, emphasizing the importance of exploring alternative treatment modalities to improve patient outcomes in COAD management.

## Supplemental data

**Table S1:** Comparison of characteristics of the COAD patients with high and low *ARL3* expression

**Parameter**	**N**	**ARL3-high**	**ARL3-low**	***P* value**
*Status*			
Alive	353	186	167	
Dead	102	42	60	0.053
*Gender*			
Female	215	104	111	
Male	240	124	116	0.543
*Race*			
Asian	11	6	5	
Black	60	24	36	
White	212	118	94	0.099
*T stage*			
T1	11	10	1	
T2	77	44	33	
T3	310	144	166	
T4	28	13	15	
T4a	19	12	7	
T4b	9	5	4	
Tis	1		1	0.034
*N stage*			
N0	268	137	131	
N1	73	35	38	
N1a	15	14	1	
N1b	15	7	8	
N1c	2	1	1	
N2	61	22	39	
N2a	8	4	4	
N2b	13	8	5	0.017
*M stage*			
M0	333	170	163	
M1	52	20	32	
M1a	9	5	4	
M1b	3	1	2	
MX	51	27	24	0.474
*TNM stage*			
I	74	45	29	
II	30	15	15	
IIA	136	66	70	
IIB	10	5	5	
IIC	1	1		
III	20	3	17	
IIIA	8	6	2	
IIIB	59	31	28	
IIIC	41	22	19	
IV	45	16	29	
IVA	17	9	8	
IVB	2	1	1	
IA	1		1	0.034

## Data Availability

The datasets used and/or analyzed during the current study are available from the corresponding author on reasonable request.
